# 1-Prop-2-ynyl-1*H*-benzimidazol-2-amine

**DOI:** 10.1107/S1600536811042772

**Published:** 2011-11-05

**Authors:** Alka Agarwal, Manavendra K. Singh, Satish K. Awasthi

**Affiliations:** a Department of Medicinal Chemistry, Institute of Medical Sciences, Banaras Hindu University, Varanasi 225 001, UP, India; b Chemical Biology Laboratory, Department of Chemistry, University of Delhi, Delhi 110 007, India

## Abstract

In the title compound, C_10_H_9_N_3_, the benzimidazol-2-amine and CH_2_—C CH units are not coplanar, with a dihedral angle of 60.36° between their mean planes. The crystal structure is stabilized by inter­molecular N—H⋯N hydrogen bonding and π–π inter­actions [centroid–centroid distances 3.677 (1) and 3.580 (1) Å], assembling the mol­ecules into a supra­molecular structure with a three-dimensional network.

## Related literature

For the biological activities of benzimidazoles, see: Nawrocka *et al.* (1999 ▶); Cuberens & Contijoch (1997 ▶); Mor *et al.* (2004 ▶); de Dios *et al.* (2005 ▶). For polyfunctionality and anti­viral activity of 2-amino­benzimidazoles, see: Garuti & Roberti (2002 ▶); Andries *et al.* (2003 ▶). For anti­proliferative properties, see: Garuti *et al.* (1998 ▶); Nawrocka *et al.* (2005 ▶). For inhibition activity against various strains of bacteria, fungi and yeasts, see: Nofal *et al.* (2002 ▶); Omar *et al.* (1996 ▶); Del Poenta *et al.* (1999 ▶). For structural analysis of small mol­ecules, see: Singh, Agarwal, Mahawar & Awasthi (2011 ▶); Singh, Singh *et al.* (2011 ▶); Singh, Agarwal & Awasthi (2011 ▶); Agarwal *et al.* (2011 ▶). For the synthesis, see: Lilienkampf *et al.* (2009 ▶).
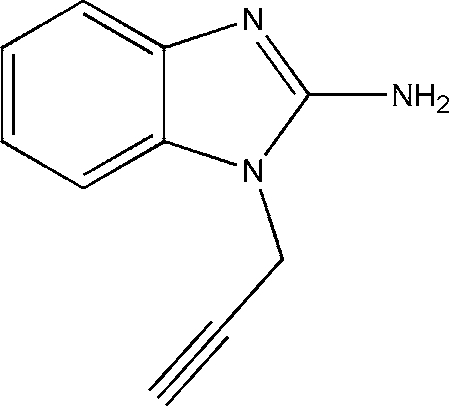


## Experimental

### 

#### Crystal data

C_10_H_9_N_3_
*M* *_r_* = 171.20
Monoclinic, 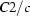

*a* = 15.385 (2) Å
*b* = 12.1433 (12) Å
*c* = 9.4653 (10) Å
β = 95.755 (11)°
*V* = 1759.5 (3) Å^3^
*Z* = 8
Mo *K*α radiation
μ = 0.08 mm^−1^
*T* = 293 K
0.39 × 0.36 × 0.20 mm

#### Data collection

Oxford Diffraction Xcalibur Sapphire3 diffractometer
Absorption correction: multi-scan (*CrysAlis PRO*; Oxford Diffraction, 2009 ▶) *T*_min_ = 0.677, *T*_max_ = 1.000
9853 measured reflections
3178 independent reflections
2505 reflections with *I* > 2σ(*I*)
*R*_int_ = 0.020

#### Refinement

*R*[*F* ^2^ > 2σ(*F* ^2^)] = 0.054
*wR*(*F* ^2^) = 0.137
*S* = 1.08
3178 reflections
126 parameters
H atoms treated by a mixture of independent and constrained refinement
Δρ_max_ = 0.25 e Å^−3^
Δρ_min_ = −0.18 e Å^−3^

### 

Data collection: *CrysAlis PRO* (Oxford Diffraction, 2009 ▶); cell refinement: *CrysAlis PRO*; data reduction: *CrysAlis PRO*; program(s) used to solve structure: *SHELXS97* (Sheldrick, 2008 ▶); program(s) used to refine structure: *SHELXL97* (Sheldrick, 2008 ▶); molecular graphics: *Mercury* (Macrae *et al.*, 2006 ▶); software used to prepare material for publication: *publCIF* (Westrip, 2010 ▶).

## Supplementary Material

Crystal structure: contains datablock(s) I, global. DOI: 10.1107/S1600536811042772/zj2027sup1.cif

Structure factors: contains datablock(s) I. DOI: 10.1107/S1600536811042772/zj2027Isup2.hkl

Supplementary material file. DOI: 10.1107/S1600536811042772/zj2027Isup3.cml

Additional supplementary materials:  crystallographic information; 3D view; checkCIF report

## Figures and Tables

**Table 1:** Hydrogen-bond geometry (Å, °)

*D*—H⋯*A*	*D*—H	H⋯*A*	*D*⋯*A*	*D*—H⋯*A*
N3—H1N3⋯N1^i^	0.90 (2)	2.36 (2)	3.1823 (18)	153.5 (18)
N3—H2N3⋯N1^ii^	0.860 (18)	2.241 (19)	3.0591 (15)	158.6 (15)

Symmetry codes: (i) 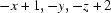; (ii) 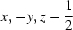.

**Fig. 1.**
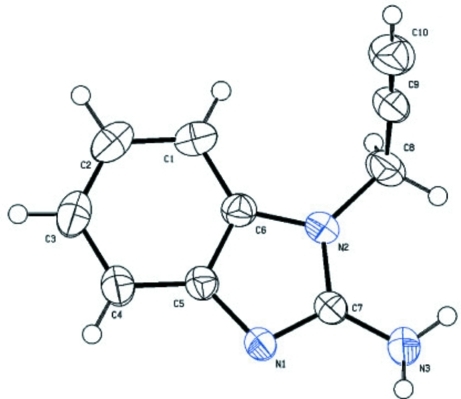
ORTEP diagram of the molecule with thermal ellipsoids drawn at 50% probability level Color code: White: C; blue: N; white: H.

**Fig. 2.**
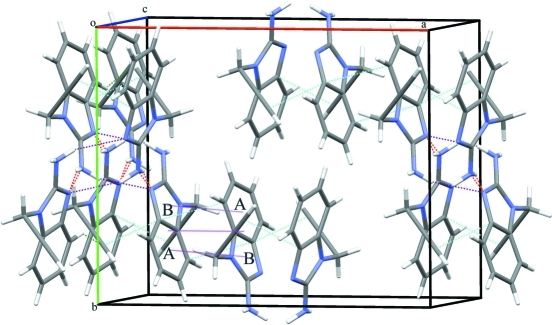
Packing diagram of molecule viewed through a plane showing supramolecule arrangement and intermolecular N—H···N (red and purple doted line) hydrogen bonding, C—H···C (Green doted line) interactions and π···π (magneta and light blue doted line) interaction.

**Fig. 3.**
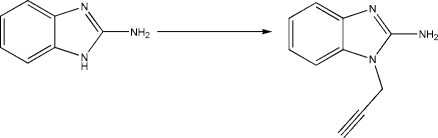
The synthesis of the title compound.

## supplementary crystallographic information

### Comment

The 2-aminobenzimidazole compounds showed different biological activities such as immunotropic, diuretic, antihistamine as well as highly selective p38a MAP inhibition properties ( Nawrocka *et al.*, 1999, Cuberens & Contijoch 1997, Mor *et al.*, 2004, De Dios *et al.*, 2005). The polyfunctionality of the 2-aminobenzimidazole molecule is due to the cyclic guanidine moiety which acts as a building block for the synthesis of a large number of benzimidazole derivatives. The various benzimidazole derivatives such as enviradene and enviroxime and other 2-aminobenzimidazole derivatives have been synthesized and screened for antiviral activity (Garuti & Roberti 2002, Andries *et al.*, 2003). Moreover, a number of 2-aminobenzimidazoles have exhibited antiproliferative properties (Garuti, Varoli *et al.*, 1998, Nawrocka *et al.*, 2005). Further, different substituted 2-aminobenzimidazoles have been found to possess inhibition activity against various strains of bacteria, fungi and yeasts in vivo and in vitro ( Nofal *et al.*, 2002, Omar *et al.*, 1996, Del Poenta *et al.*, 1999). In continuation with our recent work on structural analysis of small molecule (Singh, Agarwal, Mahawar & Awasthi 2011, Singh, Singh, Agarwal, Hussain & Awasthi 2011, Singh, Agarwal & Awasthi 2011, Agarwal *et al.*, 2011), we wish to report here the crystal structure of 1-prop-2-ynyl-1H-benzimidazol-2-ylamine (Figure1).

In the title compound, the C7-N3 single bond length (1.35 Å) is shorter than normal C-N (1.47 Å) bond suggesting a delocalized double bond in benzimidazole moiety. Again, in the crystal structure, the title compound is stabilized by intermolecular N-H···N hydrogen bonding and π···π interaction assembling, thus forming into supramolecule type assembly with a tri-dimensional network (Figure 2, Table 1). π···π interactions form a dimer, the ring A and B of an benzimidazole skeleton stacks with the ring B and A of another adjacent benzimidazole skeleton, respectively. The distance of CgA and CgB is 3.641 Å, where CgA and CgB are the center of ring A and B, respectively and the centroid - centroid distance between two adjacent benzimidazole ring is 3.580 Å and the bond distance between atoms C5···C10 is found to be 3.282 Å as well as the bond distance between atoms C1···C5 is found to be 3.369 Å, which helps in overall crystal structure packing as well as stabilization (Figure 2). In the molecule, 2-aminobenzimidazole ring and CH_2_-C≡CH are non-planar with dihedral angle is 60.36. The CCDC No. of crystal is 843876.

### Experimental

The synthesis of the title compound was carried out according to the published procedure (Lilienkampf *et al.*, 2009). Briefly, to a solution of 2- aminobenzimidazole (0.50 g m, 3.76 mmol) in dry acetone was added anhydrous K_2_CO_3_ (3.112 g m, 22.55 mmol) and reaction mixture was further refluxed for 15–30 minutes. Subsequently, KI (0.312 g m, 1.88 mmol) and propargyl bromide (0.39 ml, 4.37 mmol) were added into it and further refluxed the reaction mixture for 18 hrs. After this period, the reaction mixture was cooled, filtered and the filtrate was concentrated *invacuo* up to 10 ml and left for few days at room temperature which gave transparent crystals after slow evaporation of solvent. Yield = 30%, MS (Macromass G) m/*z* = 188 (*M*^+^), *R*_f_0.59 (98:2, CH_2_Cl_2_: MeOH) Elemental analysis (Perkin –Elmer 240°C elemental analyzer)Calculated for: C_10_ H_9_ N_3_(%) C– 70.2, H-5.3, N -24.5 found C-69.9, H-5.5, N -24.6. ^1^H NMR (CDCl_3_), 7.71–7.68 (m, 1H), 7.44–7.42 (m, 1H), 7.26–7.21 (m, 1H), 7.07–7.02 (m, 1H), 4.88 (s, 2H, NH_2_), 4.71 (s, 2H, CH_2_), 2.43 (s, 1H, CH).

### Refinement

All H atoms were located from difference Fourier map (range of C—H = 0.93 - 0.97 Å and N—H = 0.86 (18) - 0.90 (2) Å) and allowed to refine freely.

### Crystal data

**Table:**

C_10_H_9_N_3_	*F*(000) = 720.0
*M**_r_* = 171.20	*D*_x_ = 1.293 Mg m^−^^3^
Monoclinic, *C*2/*c*	Mo *K*α radiation, λ = 0.71073 Å
Hall symbol: -C 2yc	Cell parameters from 2285 reflections
*a* = 15.385 (2) Å	θ = 3.3–29.2°
*b* = 12.1433 (12) Å	µ = 0.08 mm^−^^1^
*c* = 9.4653 (10) Å	*T* = 293 K
β = 95.755 (11)°	Block, clear white
*V* = 1759.5 (3) Å^3^	0.39 × 0.36 × 0.20 mm
*Z* = 8	

### Data collection

**Table:**

Oxford Diffraction Xcalibur Sapphire3 diffractometer	3178 independent reflections
Radiation source: fine-focus sealed tube	2505 reflections with *I* > 2σ(*I*)
graphite	*R*_int_ = 0.020
ω scans	θ_max_ = 32.5°, θ_min_ = 3.0°
Absorption correction: multi-scan (*CrysAlis PRO*; Oxford Diffraction, 2009)	*h* = −19→23
*T*_min_ = 0.677, *T*_max_ = 1.000	*k* = −18→18
9853 measured reflections	*l* = −13→14

### Refinement

**Table:**

Refinement on *F*^2^	Primary atom site location: structure-invariant direct methods
Least-squares matrix: full	Secondary atom site location: difference Fourier map
*R*[*F*^2^ > 2σ(*F*^2^)] = 0.054	Hydrogen site location: inferred from neighbouring sites
*wR*(*F*^2^) = 0.137	H atoms treated by a mixture of independent and constrained refinement
*S* = 1.08	*w* = 1/[σ^2^(*F*_o_^2^) + (0.0598*P*)^2^ + 0.678*P*] where *P* = (*F*_o_^2^ + 2*F*_c_^2^)/3
3178 reflections	(Δ/σ)_max_ = 0.006
126 parameters	Δρ_max_ = 0.25 e Å^−^^3^
0 restraints	Δρ_min_ = −0.18 e Å^−^^3^

### Special details

**Table:**

Geometry. All e.s.d.'s (except the e.s.d. in the dihedral angle between two l.s. planes) are estimated using the full covariance matrix. The cell e.s.d.'s are taken into account individually in the estimation of e.s.d.'s in distances, angles and torsion angles; correlations between e.s.d.'s in cell parameters are only used when they are defined by crystal symmetry. An approximate (isotropic) treatment of cell e.s.d.'s is used for estimating e.s.d.'s involving l.s. planes.
Refinement. Refinement of *F*^2^ against ALL reflections. The weighted *R*-factor *wR* and goodness of fit *S* are based on *F*^2^, conventional *R*-factors *R* are based on *F*, with *F* set to zero for negative *F*^2^. The threshold expression of *F*^2^ > σ(*F*^2^) is used only for calculating *R*-factors(gt) *etc*. and is not relevant to the choice of reflections for refinement. *R*-factors based on *F*^2^ are statistically about twice as large as those based on *F*, and *R*- factors based on ALL data will be even larger.

### Fractional atomic coordinates and isotropic or equivalent isotropic displacement parameters (Å^2^)

**Table:**

	*x*	*y*	*z*	*U*_iso_*/*U*_eq_	
H2N3	0.4017 (11)	−0.0261 (14)	0.750 (2)	0.056 (5)*	
H1N3	0.4441 (14)	−0.0599 (17)	0.892 (2)	0.082 (6)*	
N2	0.35287 (7)	0.16350 (8)	0.82062 (9)	0.0344 (2)	
N1	0.40855 (7)	0.10067 (8)	1.03481 (10)	0.0370 (2)	
C5	0.38413 (7)	0.21017 (9)	1.04715 (11)	0.0327 (2)	
C7	0.38975 (8)	0.07726 (9)	0.89807 (11)	0.0347 (2)	
N3	0.40152 (10)	−0.02285 (9)	0.84084 (12)	0.0507 (3)	
C6	0.34953 (7)	0.25096 (9)	0.91465 (11)	0.0332 (2)	
C4	0.39113 (9)	0.27854 (11)	1.16533 (13)	0.0416 (3)	
H4	0.4128	0.2525	1.2544	0.050*	
C9	0.38623 (9)	0.24521 (11)	0.60031 (12)	0.0446 (3)	
C3	0.36468 (10)	0.38702 (11)	1.14576 (15)	0.0495 (3)	
H3	0.3700	0.4350	1.2227	0.059*	
C8	0.32884 (9)	0.16969 (11)	0.66794 (12)	0.0448 (3)	
H8A	0.2689	0.1947	0.6500	0.054*	
H8B	0.3326	0.0969	0.6268	0.054*	
C1	0.32143 (9)	0.35828 (10)	0.89445 (15)	0.0439 (3)	
H1	0.2979	0.3839	0.8062	0.053*	
C2	0.33019 (10)	0.42580 (10)	1.01311 (17)	0.0508 (3)	
H2	0.3126	0.4989	1.0040	0.061*	
C10	0.43272 (11)	0.30823 (14)	0.55223 (17)	0.0580 (4)	
H10	0.4697	0.3583	0.5140	0.070*	

### Atomic displacement parameters (Å^2^)

**Table:**

	*U*^11^	*U*^22^	*U*^33^	*U*^12^	*U*^13^	*U*^23^
N2	0.0425 (5)	0.0342 (5)	0.0259 (4)	0.0015 (4)	0.0010 (3)	0.0029 (3)
N1	0.0490 (6)	0.0362 (5)	0.0257 (4)	0.0108 (4)	0.0030 (4)	0.0004 (3)
C5	0.0345 (5)	0.0338 (5)	0.0301 (5)	0.0030 (4)	0.0054 (4)	0.0003 (4)
C7	0.0417 (6)	0.0349 (5)	0.0277 (5)	0.0055 (4)	0.0048 (4)	0.0016 (4)
N3	0.0769 (9)	0.0425 (6)	0.0319 (5)	0.0186 (6)	0.0013 (5)	−0.0051 (4)
C6	0.0347 (5)	0.0322 (5)	0.0331 (5)	−0.0005 (4)	0.0051 (4)	0.0026 (4)
C4	0.0477 (7)	0.0439 (6)	0.0336 (5)	0.0033 (5)	0.0055 (5)	−0.0059 (5)
C9	0.0586 (8)	0.0469 (7)	0.0289 (5)	0.0108 (6)	0.0069 (5)	0.0047 (5)
C3	0.0562 (8)	0.0403 (6)	0.0534 (8)	0.0005 (5)	0.0118 (6)	−0.0139 (6)
C8	0.0554 (8)	0.0482 (7)	0.0289 (5)	−0.0018 (6)	−0.0051 (5)	0.0047 (5)
C1	0.0481 (7)	0.0341 (5)	0.0491 (7)	0.0021 (5)	0.0029 (5)	0.0077 (5)
C2	0.0554 (8)	0.0308 (5)	0.0669 (9)	0.0035 (5)	0.0099 (7)	−0.0020 (6)
C10	0.0669 (10)	0.0587 (8)	0.0513 (8)	0.0076 (7)	0.0205 (7)	0.0105 (7)

### Geometric parameters (Å, °)

**Table:**

N2—C7	1.3684 (14)	C4—H4	0.9300
N2—C6	1.3899 (14)	C9—C10	1.170 (2)
N2—C8	1.4573 (14)	C9—C8	1.4641 (19)
N1—C7	1.3284 (14)	C3—C2	1.395 (2)
N1—C5	1.3898 (14)	C3—H3	0.9300
C5—C4	1.3885 (16)	C8—H8A	0.9700
C5—C6	1.4028 (15)	C8—H8B	0.9700
C7—N3	1.3505 (15)	C1—C2	1.386 (2)
N3—H2N3	0.860 (18)	C1—H1	0.9300
N3—H1N3	0.90 (2)	C2—H2	0.9300
C6—C1	1.3803 (16)	C10—H10	0.9300
C4—C3	1.3856 (19)		
			
C7—N2—C6	106.36 (9)	C5—C4—H4	121.2
C7—N2—C8	128.39 (10)	C10—C9—C8	176.81 (15)
C6—N2—C8	125.00 (10)	C4—C3—C2	121.42 (12)
C7—N1—C5	104.62 (9)	C4—C3—H3	119.3
C4—C5—N1	129.79 (11)	C2—C3—H3	119.3
C4—C5—C6	120.06 (10)	N2—C8—C9	111.21 (11)
N1—C5—C6	110.13 (9)	N2—C8—H8A	109.4
N1—C7—N3	123.96 (11)	C9—C8—H8A	109.4
N1—C7—N2	113.34 (10)	N2—C8—H8B	109.4
N3—C7—N2	122.63 (10)	C9—C8—H8B	109.4
C7—N3—H2N3	117.1 (11)	H8A—C8—H8B	108.0
C7—N3—H1N3	110.8 (13)	C6—C1—C2	116.27 (12)
H2N3—N3—H1N3	116.2 (17)	C6—C1—H1	121.9
C1—C6—N2	131.60 (11)	C2—C1—H1	121.9
C1—C6—C5	122.87 (11)	C1—C2—C3	121.79 (12)
N2—C6—C5	105.52 (9)	C1—C2—H2	119.1
C3—C4—C5	117.57 (12)	C3—C2—H2	119.1
C3—C4—H4	121.2	C9—C10—H10	180.0
			
C7—N1—C5—C4	−178.09 (12)	N1—C5—C6—C1	−178.69 (11)
C7—N1—C5—C6	0.59 (13)	C4—C5—C6—N2	179.14 (10)
C5—N1—C7—N3	−178.29 (13)	N1—C5—C6—N2	0.31 (12)
C5—N1—C7—N2	−1.34 (14)	N1—C5—C4—C3	177.22 (12)
C6—N2—C7—N1	1.56 (14)	C6—C5—C4—C3	−1.35 (18)
C8—N2—C7—N1	176.05 (11)	C5—C4—C3—C2	1.6 (2)
C6—N2—C7—N3	178.56 (12)	C7—N2—C8—C9	−110.26 (14)
C8—N2—C7—N3	−7.0 (2)	C6—N2—C8—C9	63.27 (15)
C7—N2—C6—C1	177.81 (13)	C10—C9—C8—N2	−27 (3)
C8—N2—C6—C1	3.1 (2)	N2—C6—C1—C2	−177.87 (12)
C7—N2—C6—C5	−1.07 (12)	C5—C6—C1—C2	0.85 (18)
C8—N2—C6—C5	−175.79 (11)	C6—C1—C2—C3	−0.6 (2)
C4—C5—C6—C1	0.14 (18)	C4—C3—C2—C1	−0.6 (2)

### Hydrogen-bond geometry (Å, °)

**Table:**

*D*—H···*A*	*D*—H	H···*A*	*D*···*A*	*D*—H···*A*
N3—H1N3···N1^i^	0.90 (2)	2.36 (2)	3.1823 (18)	153.5 (18)
N3—H2N3···N1^ii^	0.860 (18)	2.241 (19)	3.0591 (15)	158.6 (15)(2)

Symmetry codes: (i) −*x*+1, −*y*, −*z*+2; (ii) *x*, −*y*, *z*−1/2.
